# Supplementary material for: Relationship between family functioning, differentiation of self and anxiety in Spanish young adults
Source: PLoS One. 2021 Mar 3;16(3):e0246875. doi: 10.1371/journal.pone.0246875 (PMC7928452; doi:10.1371/journal.pone.0246875)
Supplement: Attachment — Submitted filename: Dear colleagues.docx [file pone.0246875.s002.docx]

Dear colleagues,

My name is Jesús Oliver and I´m writing you regarding the manuscript PONE-D-20-02204, that was rejected by the editor Angel Blanch, who considered that it was similar to the article PONE-D-20-02205 we sent you simultaneously, and that it was rejected too.

I´d like to point out that the authors of these studies are different (Blanca Dolz & Jesús Oliver; Marta Mozas & Jesús Oliver); the variables assessed differ too (family functioning and anxiety; parenting styles and marital satisfaction) and the samples are not de same (185 subjects; 140 subjects).

Although the editor recommended us to encapsulate both manuscripts into a single article, the authors of the second article have decided to publish it in a Spanish journal. However, the authors of the first article (PONE-D-20-02204) would like to publish this manuscript in Plos One, so we are sending you again with some corrections (PONE-D-20-20447).

We hope you find the study interesting.

Kind regards,

Jesús Oliver Pece
