# Supplementary material for: Relationship between family functioning, differentiation of self and anxiety in Spanish young adults
Source: PLoS One. 2021 Mar 3;16(3):e0246875. doi: 10.1371/journal.pone.0246875 (PMC7928452; doi:10.1371/journal.pone.0246875)
Supplement: Attachment — Submitted filename: renamed_eaed1.docx [file pone.0246875.s003.docx]

**COMMENTS – Manuscript PONE-D-20-20447**

Title: “Relationship between Family Functioning, Differentiation of Self and Anxiety

in Young Adults”

**Important note: This review pertains only to ‘statistical aspects’ of the study and so** **‘****clinical aspects’ [like medical importance,** **relevance of the study,** **‘clinical significance and** **implication(s)’ of the whole study, etc.] are to be evaluated [should be assessed] separately/independently. Further please note that any ‘statistical review’ is generally done under the assumption that (such) study specific methodological [as well as execution] issues are perfectly taken care of by the investigator(s). This review is not an exception to that and so does not cover clinical aspects {however, seldom comments are made only if those issues are intimately / scientifically related & intermingle with ‘statistical aspects’ of the study}. Agreed that ‘statistical methods’ are used as just tools here, however, they are vital part of methodology [and so should be given due importance].**

**COMMENTS:** Your ABSTRACT is well drafted but assay type. Please note that it is preferable [though this article type is ‘Research Article’] to divide the ABSTRACT with small sections like ‘Objective(s)’, ‘Methods’, ‘Results’, ‘Conclusions’, etc. [refer to item 1b of CONSORT checklist 2010: Structured summary of trial design, methods, results, and conclusions] which is an accepted practice of most good/standard journals [including PLOS-ONE]. It will definitely be more informative then, I guess, whatever the article type may be.

Though measures/tools used are appropriate, most of them yield data that are in [at the most] ‘ordinal’ level of measurement [and not in ratio level of measurement for sure {as the score two times higher does not indicate presence of that parameter/phenomenon as double (for example, a Visual Analogue Scales VAS score or say ‘depression’ score)}]. The Family Adaptability and Cohesion Evaluation Scale-20Esp (FACES - 20Esp), the Differentiation of Self Scale (EDS) and the State-Trait Anxiety Inventory (STAI) are no exception. Application/use of suitable non-parametric test(s) is/are indicated/advisable [even if distribution may be ‘Gaussian’ (i.e. normal)] in such cases.

Remember that “Inferential statistics (i.e. hypothesis testing + estimation of CI) is built on the population model (i.e. the underlying assumption is that there is a population and we are dealing with random sample(s) drawn from that population). Although in clinical trial (involving at least two groups) we do not really deal with random samples (generally a non-probabilistic convenience sampling), ‘allocation’ to treatment groups is ‘randomly’ done which enable us to evoke the population model and we can use inferential statistics safely. But when there is only one group (so that there is no question of random allocation), with ‘non-random’ selection, it may be questionable to use inferential statistics. For a pilot study it is alright to have ‘single-arm design’, or when that is the only possibility’. By quoting this I am not suggesting ‘Do not use any inferential statistics here’, however, it is very essential to keep the limitations in mind while interpreting results.

Further note that highly significant (large) value of ‘Pearson’s correlation coefficient’ alone does not imply cause-effect relationship. There are other certain criteria which are to be considered before making any causal inference(s). Statistical test usually used to assess significance of Pearson’s ‘Correlation coefficient (r)’ is ‘t’ [where t = { r √ [(n-2) / (1-r2)] }for df=n-2, n is sample size] and here Ho is that the population/standard value of ‘r’ is zero. You need r=0.878 to be significant at 5% when n=5 but you need r=0.273 if n=50 & you need only r=0.088 if n=500. ‘P-value’ heavily depends on sample size. Therefore, it is customary to use the (available in most text books on ‘Biostatistics’ or on ‘www/net’) guidelines for interpreting positive or negative correlations (and do not rely only on corresponding ‘P’-value but also consider an absolute value of ‘Correlation coefficient’).

In addition, also note that ‘Logistic regression’ is (though semi-parametric) considered as non-parametric [for all practical purposes]. Therefore, many times it is preferred oven ‘Simple/Multiple Linear Regression’ {because often few ‘independent’ variables are not continuous as required (strictly assumed as continuous) by ‘Multiple Linear Regression’}. Completely (pure/exclusive) non-parametric regression though available, is not very popular [and so software is not available for estimation (in my knowledge)]. Therefore, not necessary to go for ‘non-parametric regression’ and ‘Logistic regression’ is enough to apply here. However, note that [as pointed out in ‘limitations’ (first limitation) on page 23], this design (cross-sectional survey) do not allow to indicate cause-effect relationships between the different variables studied.

In context of ‘The Differentiation of Self Scale (DSS) given on page 12, please note the following:

Whenever response options ranged from 1=strongly disagree to 4=strongly agree (or ranging from 1 (strongly disagree) to 6 (strongly agree)), while using a ‘Likert’ scale responses, recoding [like strongly disagree=-2, disagree=-1, neutral=0, agree=1, strongly agree=2] may yield correct and meaningful ‘arithmetic mean’ which is useful not only for comparison but has absolute meaning, in my opinion. Application of any statistical test(s) assume that meaning of entity used (mean, SD, etc) has a particular meaning. However, ‘α’ [alpha] or most other measures of reliability/correlation will remain same.

I believe that the ‘F’ value given in table-2 [Table 2: *Difference of Means between* *Women and Men in the Differentiation of Self subscales*] is from multivariate analysis of variance (MANOVA) as said in text. When only two groups are to be compared {ex. Women and Men}, we use ‘t’ test for two independent groups [non-parametric equivalent to unpaired ‘t’ test is Mann-Whitney ‘U’ test] and not ANOVA ‘F’ [definitely/certainly not ‘multivariate’ ANOVA called ‘MANOVA’]. Although ‘F’ and ‘t’ are mathematically related/equivalent [square of ‘t’ is exactly equal to ‘F’ if (mistakenly) calculated for two groups], logic/philosophy (and so underlying assumptions) behind their development and algorithms used for estimation of test statistic are different. They are applicable in different situations.

[in table-2, for ‘total’ mean=4.17, SD=0.49, n=123 for ‘Women’ group and mean=4.33, SD=0.41, n=62 for ‘Men’ group then ‘t’=2.21. Repored is F=7.190 (which is unfortunately not just square of 2.21, may be because this ‘F’ is yielded by ‘multivariate MANOVA’ and not ‘univariate ANOVA’). Mind you that this is a scientific/academic document and so should be reported only scientifically correct, appropriate things.]

Moreover, what is ‘Lambda’ in table-2. Interpretation of ‘Lambda=0.833’ is not given anywhere. You may know that Wilks’ lambda is ‘pooled ratio of error variances to effect variance plus error variance’. My question is “what is the role of ‘Lambda’ value here”? Simillarly, I could not understand [refer to second ‘limitation’ on page 23] “When you said ‘the sample has had a greater representation of women than men, which has made it difficult to perform analysis of comparison of means between groups” How was that? Please explain.

Overall the study has potential but needs ‘major revision’, in my opinion. Introduction and description/explanation of other concepts [like Differentiation of Self] are nice.
