# Supplementary material for: Relationship between family functioning, differentiation of self and anxiety in Spanish young adults
Source: PLoS One. 2021 Mar 3;16(3):e0246875. doi: 10.1371/journal.pone.0246875 (PMC7928452; doi:10.1371/journal.pone.0246875)
Supplement: Attachment — Submitted filename: +++Response to reviewers B. docx.docx [file pone.0246875.s004.docx]

**Comments to the Author**

1. Is the manuscript technically sound, and do the data support the conclusions?

The manuscript must describe a technically sound piece of scientific research with data that supports the conclusions. Experiments must have been conducted rigorously, with appropriate controls, replication, and sample sizes. The conclusions must be drawn appropriately based on the data presented.

Reviewer #1: Partly

Reviewer #2: Partly

The article insists that the results are limited to our Spanish sample and it indicates as a limitation the small sample size. Nevertheless, after the new revision, we think that the study has been conducted rigorously, contains interesting results and the discussion points out that the results coincide with Bowen´s postulates and with those obtained in other studies carried out in other countries as well as in Spain.

2. Has the statistical analysis been performed appropriately and rigorously?

Reviewer #1: N/A

Reviewer #2: No

Taking into account your suggestion, we substituted the MANOVA test for several Student´s t test. After the new revision, we think that all the statistical analysis have been performed appropriately and rigorously.

3. Have the authors made all data underlying the findings in their manuscript fully available?

The [PLOS Data policy](http://www.plosone.org/static/policies.action#sharing) requires authors to make all data underlying the findings described in their manuscript fully available without restriction, with rare exception (please refer to the Data Availability Statement in the manuscript PDF file). The data should be provided as part of the manuscript or its supporting information, or deposited to a public repository. For example, in addition to summary statistics, the data points behind means, medians and variance measures should be available. If there are restrictions on publicly sharing data—e.g. participant privacy or use of data from a third party—those must be specified.

Reviewer #1: Yes

Reviewer #2: Yes

4. Is the manuscript presented in an intelligible fashion and written in standard English?

PLOS ONE does not copyedit accepted manuscripts, so the language in submitted articles must be clear, correct, and unambiguous. Any typographical or grammatical errors should be corrected at revision, so please note any specific errors here.

Reviewer #1: Yes

Reviewer #2: Yes

5. Review Comments to the Author

Please use the space provided to explain your answers to the questions above. You may also include additional comments for the author, including concerns about dual publication, research ethics, or publication ethics. (Please upload your review as an attachment if it exceeds 20,000 characters)

**Reviewer #1:**

In the manuscript entitled "Relationship between Family Functioning, Differentiation of Self and Anxiety in Young Adults" the authors examined relations between study variables along with diference tests and a mediation test. Although the manuscript seems interesting and has the potential for the family therapy literature, I have some major concerns about the manuscript:

1. My biggest concern is about the **small sample size** for these multiple analyses. The authors used scales with 74 items, 20 items, and 40 items in addition to a demographic questionnaire. Along with a lot of items to be filled, they used multiple analyses, which makes the generability of the results very very limited. I **recommend authors to enlarge their sample**.

Thank you very much for your recommendation. We agree with you but, unfortunately, we do not have the possibility to enlarge the sample and do all the analyzes again, because we have to submit the article by December 23^th^ and we are really busy with other research projects underway and with the bimodal teaching at the university due to the pandemic in Spain. Due to the small sample size, we have insisted throughout the article that the results are limited to our Spanish sample and we have not tried to generalize them to the Spanish population. Nevertheless, we have indicated in the discussion that our results coincide with Bowen´s postulates and with those obtained in other studies carried out in other countries and in Spain.

2. My second biggest concern is that the authors do not explain their **analyses procedures** within the manuscript. For instance, how did they perform mediation analysis? Which procedures did they follow? Yes, they say that thay used macro PROCESS version 3.3for SPSS, but what about the procedures? **They should explicitly tell their analytic procedures step by step with the relevant citations for each methodology they used**.

Thank you very much for your recommendation. We have added a new section about Data analysis, where we explain that the assumptions of each test were checked, and we describe all the statistical analyses carried out in the study, and specifically, the mediation analysis and some citations that explain our procedure.

3. The authors should give more background information for the rationale of their study. In this form, they explain each variable separately, but the readers should know **how these constructs are related**. What are the potential mechanisms bringing these constructs together in a research? Thus, the authors should give these information instead of giving seperate construct explanations.

Thank you very much for your recommendation. We have included in the section **Differentiation of self** three paragraphs about Bowen´s postulates, which posit that family functioning, differentiation of self and anxiety are related. The studies about these relationships are not abundant, so we have been able to include just a few references (e.g., Chung & Gale, 2009; Duch-Ceballos et al., 2020; Isik & Bulduk, 2015; Johnson et al., 2001; Kim et al., 2014, 2015; Peleg-Popko, 2002a, 2005; Skowron & Friedlander, 1998). However, this is an important reason to perform this study.

**Reviewer #2:**

**Important note: This review pertains only to ‘statistical aspects’ of the study and so** **‘****clinical aspects’ [like medical importance,** **relevance of the study,** **‘clinical significance and** **implication(s)’ of the whole study, etc.] are to be evaluated [should be assessed] separately/independently. Further please note that any ‘statistical review’ is generally done under the assumption that (such) study specific methodological [as well as execution] issues are perfectly taken care of by the investigator(s). This review is not an exception to that and so does not cover clinical aspects {however, seldom comments are made only if those issues are intimately / scientifically related & intermingle with ‘statistical aspects’ of the study}. Agreed that ‘statistical methods’ are used as just tools here, however, they are vital part of methodology [and so should be given due importance].**

**COMMENTS:** Your ABSTRACT is well drafted but assay type. Please note that it is preferable [though this article type is ‘Research Article’] to divide the ABSTRACT with small sections like **‘Objective(s)’, ‘Methods’, ‘Results’, ‘Conclusions’,** etc. [refer to item 1b of CONSORT checklist 2010: Structured summary of trial design, methods, results, and conclusions] which is an accepted practice of most good/standard journals [including PLOS-ONE]. It will definitely be more informative then, I guess, whatever the article type may be.

Thank you very much for your recommendation. We have included the abstract with all these sections.

Though measures/tools used are appropriate, most of them yield data that are in [at the most] **‘ordinal’ level of measurement [and not in ratio level of measurement** for sure {as the score two times higher does not indicate presence of that parameter/phenomenon as double (for example, a Visual Analogue Scales VAS score or say ‘depression’ score)}]. The Family Adaptability and Cohesion Evaluation Scale-20Esp (FACES - 20Esp), the Differentiation of Self Scale (EDS) and the State-Trait Anxiety Inventory (STAI) are no exception. Application/use of suitable non-parametric test(s) is/are indicated/advisable [even if distribution may be ‘Gaussian’ (i.e. normal)] in such cases.

Thank you very much for your comment. We have followed the instructions of the instruments and they do not use standardized scores or percentiles. Also, we have followed the method used in most of the studies about family functioning and differentiation of self.

Remember that “Inferential statistics (i.e. hypothesis testing + estimation of CI) is built on the population model (i.e. the underlying assumption is that there is a population and we are dealing with random sample(s) drawn from that population). Although in clinical trial (involving at least two groups) we do not really deal with random samples (generally a non-probabilistic convenience sampling), ‘allocation’ to treatment groups is ‘randomly’ done which enable us to evoke the population model and we can use inferential statistics safely. But **when there is only one group** (so that there is no question of random allocation), **with ‘non-random’ selection, it may be questionable to use inferential statistics.** For a pilot study it is alright to have ‘single-arm design’, or when that is the only possibility’. **By quoting this I am not suggesting ‘Do not use any inferential statistics here**’, however, **it is very essential to keep the limitations in mind while interpreting results.**

Thank you very much for your comment. We have tried to be very cautious with the interpretations. So, we have pointed out in the article that the results are limited to our Spanish sample and we highlight as a limitation that the sample size of our study is limited.

Further note that **highly significant (large) value of ‘Pearson’s correlation coefficient’ alone does not imply cause-effect relationship**. There are other certain criteria which are to be considered before making any causal inference(s). Statistical test usually used to assess significance of Pearson’s ‘Correlation coefficient (r)’ is **‘t’** [where t = { r √ [(n-2) / (1-r2)] }for df=n-2, n is sample size] and here Ho is that the population/standard value of ‘r’ is zero. **You need r=0.878 to be significant at 5% when n=5 but you need r=0.273 if n=50 & you need only r=0.088 if n=500**. ‘P-value’ heavily depends on sample size. Therefore, **it is customary to use the** (available in most text books on ‘Biostatistics’ or on ‘www/net’) **guidelines for interpreting positive or negative correlations (and do not rely only on corresponding ‘P’-value but also consider an absolute value of ‘Correlation coefficient’).**

Thank you very much for your comment. As we have pointed out in the limitations of the study (on page 26), “It must be taken into account the transversal and correlational nature of the data, which do not allow us to indicate cause-effect relationships between the different variables studied, so longitudinal studies are needed to evaluate the direction of the effect between family functioning, differentiation of self and trait anxiety”. On the other hand, we have interpreted the correlation coefficients, and not the p values, using text books.

In addition, also note that ‘Logistic regression’ is (though semi-parametric) considered as non-parametric [for all practical purposes]. Therefore, **many times it is preferred oven ‘Simple/Multiple Linear Regression’** {because often few ‘independent’ variables are not continuous as required (strictly assumed as continuous) by ‘Multiple Linear Regression’}. Completely (pure/exclusive) non-parametric regression though available, is not very popular [and so software is not available for estimation (in my knowledge)]. Therefore, not necessary to go for ‘non-parametric regression’ and ‘Logistic regression’ is enough to apply here. However, note that [as pointed out in ‘limitations’ (first limitation) on page 23], this design (cross-sectional survey) **do not allow to indicate cause-effect relationships between the different variables studied.**

Thank you very much for your comment. We absolutely agree with this comment. As you have stated, we have pointed out in the limitations of the study, “It must be taken into account the transversal and correlational nature of the data, which do not allow us to indicate cause-effect relationships between the different variables studied, so longitudinal studies are needed to evaluate the direction of the effect between family functioning, differentiation of self and trait anxiety”.

In context of ‘The Differentiation of Self Scale (DSS) given on page 12, please note the following:

Whenever response options ranged from 1=strongly disagree to 4=strongly agree (or ranging from 1 (strongly disagree) to 6 (strongly agree)), while using a ‘Likert’ scale responses, **recoding [like strongly disagree=-2, disagree=-1, neutral=0, agree=1, strongly agree=2]** may yield correct and meaningful ‘arithmetic mean’ which is useful not only for comparison but has absolute meaning, in my opinion. Application of any statistical test(s) assume that meaning of entity used (mean, SD, etc) has a particular meaning. However, ‘α’ [alpha] or most other measures of reliability/correlation will remain same.

Thank you very much for your interesting comment. We have tried to follow the instructions of the instruments and the method used in most of the studies about family functioning and differentiation of self., but we will follow your suggestion in future research.

I believe that the ‘F’ value given in table-2 [Table 2: *Difference of Means between* *Women and Men in the Differentiation of Self subscales*] is from multivariate analysis of variance (MANOVA) as said in text. When only **two groups are to be compared** {ex. Women and Men}, **we use ‘t’ test for two independent groups** [non-parametric equivalent to unpaired ‘t’ test is Mann-Whitney ‘U’ test] **and not ANOVA ‘F’ [definitely/certainly not ‘multivariate’ ANOVA called ‘MANOVA’].** Although ‘F’ and ‘t’ are mathematically related/equivalent [square of ‘t’ is exactly equal to ‘F’ if (mistakenly) calculated for two groups], logic/philosophy (and so underlying assumptions) behind their development and algorithms used for estimation of test statistic are different. They are applicable in different situations.

[in table-2, for ‘total’ mean=4.17, SD=0.49, n=123 for ‘Women’ group and mean=4.33, SD=0.41, n=62 for ‘Men’ group then **‘t’=2.21. Reported is F=7.190** (which is unfortunately not just square of 2.21, may be because this ‘F’ is yielded by ‘**multivariate MANOVA’ and not ‘univariate ANOVA**’). Mind you that this is a scientific/academic document and so should be reported only scientifically correct, appropriate things.]

Moreover, what is ‘Lambda’ in table-2. Interpretation of **‘Lambda=0.833’ is not given anywhere. You may know that Wilks’ lambda is ‘pooled ratio of error variances to effect variance plus error variance’. My question is “what is the role of ‘Lambda’ value here”?**

Thank you very much for your interesting comment. We thought that MANOVA test was the proper test to check differences in some variables that belong to the same construct. Taking into account your suggestion, we have substituted the MANOVA test for several Student´s t test.

Simillarly, I could not understand [refer to second ‘limitation’ on page 23] “When you said **‘the sample has had a greater representation of women than men, which has made it difficult to perform analysis of comparison of means between groups” How was that? Please explain**.

Thank you very much for your comment We are sorry, it was a misunderstanding. We have deleted the sentence “, which has made it difficult to perform analysis of comparison of means between groups”.

Overall **the study has potential but needs ‘major revision’, in my opinion.** Introduction and description/explanation of other concepts [like Differentiation of Self] are nice.

Thank you very much for your recommendation. We have tried to revise all the manuscript taking into account all your comments. We hope you find it suitable.

 6. PLOS authors have the option to publish the peer review history of their article ([what does this mean?](https://journals.plos.org/plosone/s/editorial-and-peer-review-process#loc-peer-review-history)). If published, this will include your full peer review and any attached files.


If you choose “no”, your identity will remain anonymous but your review may still be made public.

**Do you want your identity to be public for this peer review?** For information about this choice, including consent withdrawal, please see our [Privacy Policy](https://www.plos.org/privacy-policy).

Reviewer #1: No

Reviewer #2: No
