# Supplementary material for: Relationship between family functioning, differentiation of self and anxiety in Spanish young adults
Source: PLoS One. 2021 Mar 3;16(3):e0246875. doi: 10.1371/journal.pone.0246875 (PMC7928452; doi:10.1371/journal.pone.0246875)
Supplement: Attachment — Submitted filename: New comments on PONE-D-20-20447R1.docx [file pone.0246875.s005.docx]

**Manuscript Number:** PONE-D-20-20447R1
**Manuscript Title:** Relationship between Family Functioning, Differentiation of Self and Anxiety in Spanish Young Adults

**COMMENTS:** Since for most of the comments made on earlier draft by me (and by other respected reviewer also) a general response is “We have tried to follow the instructions of the instruments and the method used in most of the studies about family functioning and differentiation of self [although it is said sometimes that “we will follow your suggestion in future research”], I am confused now about any recommendation. However, I see that the manuscript is improved.

Nevertheless, now I see few different issues/queries: in table-2, ‘what is “d” [last column, no.8]?’ is not explained. I believe, ‘d’ is ‘effect size’, an (useful) index given by Cohen. If so, ‘why it is not described properly?’. At few places kindly check for the ‘English’ language {example, 2^nd^ para below table-2 you say ‘linear regression model was performed ‘. Is model performed? Or applied/used?}. Agreed, that English is not our mother tongue however, remember that this is a scientific/academic document and so all details should be clearly/correctly communicated.

In same para [2^nd^ para below table-2] you have given R²=.188 {by-the-way *R²* is called as ‘coefficient of Determination’}, note that variation explained (18.8%) is not much. As I said earlier [which unfortunately still holds], “Overall the study has potential but needs ‘major revision’, in my opinion”.
