# Supplementary material for: Relationship between family functioning, differentiation of self and anxiety in Spanish young adults
Source: PLoS One. 2021 Mar 3;16(3):e0246875. doi: 10.1371/journal.pone.0246875 (PMC7928452; doi:10.1371/journal.pone.0246875)
Supplement: Attachment — Submitted filename: +++Response to reviewers II.docx [file pone.0246875.s006.docx]

Additional Editor Comments:

Considering that for PLOS ONE the type of manuscript that you have sent is a Research Article. I suggest that the authors rewrite the background or introduction section, focusing on reporting only research results and empirical findings that allow a clear answer to the research question, and that in the background section the authors show empirical evidence of the relationship between family functioning, self and anxiety. Considering that your manuscript sent to PLOS ONE is not a narrative review article of the literature, it is not necessary to include in the background section theoretical aspects such as: Systemic Theory, Circumplex Model, and Bowen’s Family Systems Theory. Or these topics could be included, in the context of the article, but not in the text, because it is an empirical article. Therefore, I suggest that the authors consider including in the introductory section only empirical evidence and recent research results on family functioning, self and anxiety. It is suggested to reduce the length of the introductory section of this manuscript. It is suggested to check the following link of Pubmed in which you can find some examples, to read and citation.

Thank you very much for your recommendation. We have tried to reduce the theoretical section, keeping the definition of the variables and the empirical review.

**Comments to the Author**

1. If the authors have adequately addressed your comments raised in a previous round of review and you feel that this manuscript is now acceptable for publication, you may indicate that here to bypass the “Comments to the Author” section, enter your conflict of interest statement in the “Confidential to Editor” section, and submit your "Accept" recommendation.

Reviewer #1: All comments have been addressed

Reviewer #2: (No Response)

2. Is the manuscript technically sound, and do the data support the conclusions?

The manuscript must describe a technically sound piece of scientific research with data that supports the conclusions. Experiments must have been conducted rigorously, with appropriate controls, replication, and sample sizes. The conclusions must be drawn appropriately based on the data presented.

Reviewer #1: Yes

Reviewer #2: (No Response)

3. Has the statistical analysis been performed appropriately and rigorously?

Reviewer #1: Yes

Reviewer #2: (No Response)

4. Have the authors made all data underlying the findings in their manuscript fully available?

The [PLOS Data policy](http://www.plosone.org/static/policies.action#sharing) requires authors to make all data underlying the findings described in their manuscript fully available without restriction, with rare exception (please refer to the Data Availability Statement in the manuscript PDF file). The data should be provided as part of the manuscript or its supporting information, or deposited to a public repository. For example, in addition to summary statistics, the data points behind means, medians and variance measures should be available. If there are restrictions on publicly sharing data—e.g. participant privacy or use of data from a third party—those must be specified.

Reviewer #1: Yes

Reviewer #2: (No Response)

5. Is the manuscript presented in an intelligible fashion and written in standard English?

PLOS ONE does not copyedit accepted manuscripts, so the language in submitted articles must be clear, correct, and unambiguous. Any typographical or grammatical errors should be corrected at revision, so please note any specific errors here.

Reviewer #1: Yes

Reviewer #2: (No Response)

6. Review Comments to the Author

Please use the space provided to explain your answers to the questions above. You may also include additional comments for the author, including concerns about dual publication, research ethics, or publication ethics. (Please upload your review as an attachment if it exceeds 20,000 characters)

Reviewer #1: After reviewing the changes made by the author(s), I can see that they have attended to the revisions appropriately and the manuscript seems acceptable to be published within this form.

Reviewer #2: COMMENTS: Since for most of the comments made on earlier draft by me (and by other respected reviewer also) a general response is “We have tried to follow the instructions of the instruments and the method used in most of the studies about family functioning and differentiation of self [although it is said sometimes that “we will follow your suggestion in future research”], I am confused now about any recommendation. However, I see that the manuscript is improved.

Thank you very much for your comment. We have considered carefully all your advice, which we appreciate very much. But in this case we have tried to follow the instructions of the instruments used in this study and the method of most research about family functioning and differentiation of self, which use parametrical tests. It is true that Likert type scales are technically ordinal because they consist of a series of ordered categories. However, several authors have found consistent support for the use of these variables as approximately continuous. Firstly, they have observed that Likert type scales, or ordinal variables with five or more categories can be used as continuous without any harm to the statistical analysis (Johnson & Creech, 1983; Norman, 2010; Sullivan & Artino, 2013; Zumbo & Zimmerman, 1993). Secondly, they argue that the total scores of the Likert type scales consist of the sums or means of severalquestions or items–ordinal variables-. The sums or the means result in a number of categories much higher than the categories of the ordinal Likert scales they were calculated from, which results in an approximately continuous variable (Norman, 2010). This is why we have performed parametric tests in this study. However, we pointed out that “we will follow your suggestion in future research” because we are constructing a new Likert scale and we will use the range of responses that you have suggested (from -3 to +3), and if most studies about a topic use non-parametric tests, we will conduct them.

**References**

Johnson, D.R., & Creech, J.C. (1983). Ordinal measures in multiple indicator models: A simulation study of categorization error. *American Sociological Review*, *48*, 398-407.[https://doi.org/10.2307/2095231](https://psycnet.apa.org/doi/10.2307/2095231)

Norman, G. (2010). Likert scales, levels of measurement and the “laws” of statistics. *Advances in Health Sciences Education*, *15*(5), 625-632. <https://doi.org/10.1007/s10459-010-9222-y>

Sullivan, G. & Artino, A. R. (2013). Analyzing and Interpreting Data From Likert-Type Scales. *Journal of Graduate Medical Education*, *5*(4), 541-542.<https://doi.org/10.4300/JGME-5-4-18>

Zumbo, B. D., & Zimmerman, D. W. (1993). Is the selection of statistical methods governed by level of measurement? *Canadian Psychology*, *34*, 390-400.[https://doi.org/10.1037/h0078865](https://doi.apa.org/doi/10.1037/h0078865)

Nevertheless, now I see few different issues/queries: in table-2, ‘what is “d” [last column, no.8]?’ is not explained. I believe, ‘d’ is ‘effect size’, an (useful) index given by Cohen. If so, ‘why it is not described properly?’.

Thank you very much for your comment. We have change the title of the last column, and the results in parentheses from “d” to “Cohen´s d”. We have also interpreted the values of all effect sizes.

At few places kindly check for the ‘English’ language {example, 2nd para below table-2 you say ‘linear regression model was performed ‘. Is model performed? Or applied/used?}. Agreed, that English is not our mother tongue however, remember that this is a scientific/academic document and so all details should be clearly/correctly communicated.

Thank you very much for your correction. We have tried to write the text properly, but it seems that we have made a mistake. We have changed the sentence from “linear regression model was performed” to “linear regression analysis was conducted”.

In same para [2nd para below table-2] you have given R²=.188 {by-the-way R² is called as ‘coefficient of Determination’}, note that variation explained (18.8%) is not much. As I said earlier [which unfortunately still holds], “Overall the study has potential but needs ‘major revision’, in my opinion”.

Thank you very much for your comments. We did not intend to find the model that best predicts differentiation of self, but to know if family functioning predicted differentiation of self and what was the predictive power, which represents a fifth of differentiation. However, following your advice, we have decided to add another result that we also found: family functioning and differentiation of self predict 50% of anxiety. We believe that this result is also relevant and may be of greater interest than the previous one.

---------------
Thank you very much for your all your recommendations. We have tried to revise all the manuscript taking into account all your comments. We hope you find it suitable.
